# Knowledge of, and Attitudes towards, Live Fish Transport among Aquaculture Industry Stakeholders in China: A Qualitative Study

**DOI:** 10.3390/ani11092678

**Published:** 2021-09-13

**Authors:** Yifei Yang, Tingyun Wang, Clive J. C. Phillips, Qingjun Shao, Edward Narayan, Kris Descovich

**Affiliations:** 1 School of Veterinary Science, The University of Queensland, Gatton, QLD 4343, Australia; yifei.yang@uq.net.au; 2 School of Agriculture and Food Sciences, The University of Queensland, Gatton, QLD 4343, Australia; t.y.wang@uq.net.au (T.W.); e.narayan@uq.edu.au (E.N.); 3 Curtin University Sustainability Policy (CUSP) Institute, Curtin University, Kent St., Bentley, WA 6102, Australia; clive.phillips@curtin.edu.au; 4 College of Animal Science, Zhejiang University, Hangzhou 310058, China; qjshao@zju.edu.cn

**Keywords:** animal welfare, fish welfare, fish, live transport, China, stakeholder, attitudes

## Abstract

**Simple Summary:**

China is the world’s largest producer of food fish, and Chinese consumers have a preference to buy live fish. Live transport of fish is, therefore, a common procedure in aquaculture and is a potential animal welfare hazard. Little has been published on current fish transportation practices in China or the knowledge and attitudes of stakeholders in this industry. Our qualitative study aimed to obtain original information about live transport processes from a cross-section of aquaculture stakeholders in China by conducting individual interviews. Stakeholders were interviewed about their knowledge of live transport and their attitudes towards the welfare of fish. Self-described knowledge of live transport varied between participants with different job types. Most participants had heard of and understood the concept of “animal welfare”, but many understood it to only refer to terrestrial livestock, not fish. This suggests that knowledge of fish welfare in the industry may be less than for other farm animals. The findings of this pilot study contribute to a better understanding of live fish transport from a stakeholder point of view. The findings will also assist in informing, educating, and sensitizing stakeholders to the importance of fish welfare during live transport.

**Abstract:**

China is the largest food fish producer in the world. Chinese consumers normally purchase fish that are still alive to ensure freshness. Therefore, the live transport of fish is important in China’s aquaculture, although it carries potential risks for animal welfare. This study investigated the attitudes and knowledge of stakeholders within Chinese aquaculture towards the live transport and welfare of fish. Semi-structured interviews were conducted with 12 participants who were involved with the aquaculture industry in China. Most participants self-rated their transport-related knowledge as moderate and had some understanding of animal welfare, although this term was generally considered only relevant to terrestrial animals. Participants’ responses indicated that the live transport of fish occurs frequently in China, generally using sealed tanks, plastic bags, and foam boxes, in purpose-built vehicles. Seasonal changes, such as changes in ambient and water temperature, are considered to be important contributors to successful live transport, as well as sufficient oxygen supplies and stocking density. The use of anesthetics was not commonly reported, particularly in food fish, and fish capture is predominantly by conventional dipnets. The health status of transported fish is determined mostly by morphology (body injury, body or eye color, and fin condition), as well as vigor and swimming ability. Our results indicate that live transport poses a number of welfare risks to fish but that participants in the process associated welfare concerns more with terrestrial animals, not fish.

## 1. Introduction

Fish is an important dietary protein for many people in the world, and global fish consumption has continuously increased over recent years [1]. With the rapid global growth of aquaculture production, like other farmed terrestrial animals, concern for the well-being of farmed fish has also gained attention among consumers, animal protection activists, researchers, and producers [2].

Road transport of live fish by vehicle (henceforth, live transport) is a common practice in aquaculture, but it can lead to detrimental effects on fish well-being. Fish are often transported between farms or to markets for on-growing or sale [3]. There are two main methods for transporting live fish in water. The first is by using water-filled containers equipped with an outside oxygen source (e.g., oxygen tanks), and the second is by using sealed plastic bags filled with oxygen prior to transport, described as the open system and the closed system, respectively [4,5]. Live transport includes pre-transport procedures (grading, crowding, netting, fasting, handling, and loading/packing), during transport procedures, and post-transport processes (unloading and handling), which are potentially stressful to fish [6,7]. Common stressors associated with live transport are inappropriate handling, air exposure, food deprivation, poor water quality, inappropriate transport densities, sudden changes in water temperature, and rapid water movement [3,5,8,9,10]. It is challenging to maintain the health and well-being of live fish during transport, particularly because of the large numbers of animals in many varied transport situations. Transported fish show physiological responses indicative of stress, such as elevated glucocorticoid (e.g., cortisol) levels and blood glucose content, and excessive physiological stress is known to reduce fish vitality and increase mortality [7,8,11].

China has led global freshwater aquaculture production since the 1990s [1,12,13]. Inland aquaculture, particularly freshwater farming of finfish, is the main component of the Chinese aquaculture industry, comprising more than 50% of the country’s total aquaculture production in 2019 [11,12]. The majority of this production is in the provinces of Hubei, Guangdong, Jiangsu, Hunan, Jiangxi, Anhui, and Zhejiang [11], which largely fall in South and East China where water is generally abundant. Cyprinid species (e.g., grass carp *Ctenopharyngodon Idella* and silver carp *Hypophthalmichthys molitrix*) are predominant in most of the production in China [2,11,12,13]. Although production methods used in Chinese freshwater aquaculture are highly diverse, pond culture is the most common rearing method [11,12].

In China, consumers prefer to purchase fish while still alive for later cooking, which is believed to be healthier for the consumer and better tasting than fish that are killed earlier or preserved [14]. Live fish from domestic markets are preferred to frozen and processed products [14,15]. A substantial proportion of aquatic products are transported between provinces in China, largely by road [12]. However, transportation and sale of live fish can have challenges for meeting health regulations, quality standards, and animal welfare requirements. The mortality of transported live fish is one of the biggest concerns for Chinese aquaculture, particularly transport companies [16]. It is estimated that around 7% of farmed fish die annually due to live transport [17], and this is attributed to the transport time, inappropriate transport procedures, and inadequate monitoring technology [14,18]. Meanwhile, freshwater fish farming is also geographically imbalanced in China [13], which increases the duration of transport time to some regions.

Animal welfare is defined as “the physical and mental state of an animal in relation to the conditions in which it lives and dies” [19], which is an important consideration for farm animal production. Animal welfare can be assessed against a variety of standards, such as the “Five Freedoms” [20], as well as the Five Domains Model, which was developed and updated to incorporate advances in animal welfare science [21]. The Five Domains Model assesses animal welfare from the perspectives of (1) nutrition, (2) physical environment, (3) health, (4) behavioral interactions, and (5) mental state [21]. In Europe, many organizations have issued standards or guidelines to improve and ensure farmed fish welfare. For example, the World Organization for Animal Health (OIE) publishes the Aquatic Animal Health Code that provides guidelines for the health, welfare, and international trade of farmed fish [22]. Similarly, the Royal Society for the Prevention of Cruelty to Animals (RSPCA) in the United Kingdom has standards for two farmed fish species—Atlantic salmon (*Salmo salar*) and rainbow trout (*Oncorhynchus mykiss*)—which includes guidelines for maintaining fish welfare during transport. China only has one national standard (GB/T 27638-2011) that contains some recommendations relevant to live fish transport [23]. This code classifies live transport into road transport with water, road transport with little or no water (waterless transport), and vessel transport [23]. For live transport with water, the code outlines procedures for selecting healthy fish, pre-transport food withdrawal (1–2 d), pre-transport stocking density (suggested to be 20–45 kg/m^3^), and acclimation, control of water quality during both acclimation and transport, transport tools and methods, oxygen supply (>8 mg/L), and maximum transport time (<40 h). Unlike in other welfare standards mentioned previously, this code does not include welfare requirements for any specific fish species, transport density, or training for transportation staff, although these are known to affect fish welfare during live transport [8,24]. Moreover, the code only generally describes live transport procedures and lacks specific descriptions, such as loading and unloading processes, responsibilities of transport staff, and other transport-related welfare issues [23]. Lack of species-specific standards or regulations of live transport may cause transport-related welfare issues for fish.

Live transport procedures for fish have been described in the English scientific literature, but most are not connected to Chinese contexts [4,5,25]. For example, the RSPCA UK issued welfare standards for Atlantic salmon [26] and rainbow trout [27], which are commonly farmed fish in Europe, but Asian carps are the most popular farmed species in China [12]. Fish welfare is starting to become a concern in China, as evidenced by the appearance of reviews on that topic since 2009 [25,28,29]. In China, current research on live fish transport published in English focuses on waterless transport techniques [11,16,30], the efficacy of fish anesthetics [31,32], transport density [30,33], and physiological stress responses of transported fish [7,34]. Although several reviews on fish transport [19,35,36] have also been written in Chinese, their contents are very similar to each other and do not connect to welfare issues.

In livestock production, industry stakeholders, including farmers and service providers (e.g., retailers and transporters), are usually responsible for the welfare of their animals, as well as responsible to consumers for food quality and safety [37]. There is a need to communicate with stakeholders to understand existing and emerging issues relating to Chinese aquaculture, particularly around live transport of fish, as there is currently limited information available. The qualitative research method is useful when limited data have been published on a topic [38], and it contributes to a rich understanding of the human condition through their various experiences and observed circumstances [39]. Grounded theory commonly uses face-to-face interviews to investigate a particular phenomenon or a less-known issue or situation [40]. Moreover, an interview allows researchers to have a deeper discussion with participants [39]. This study aimed to obtain initial information on the knowledge of Chinese stakeholders from within the aquaculture industry or working in fishery-related positions relevant to live transport procedures. We also aimed to garner an in-depth understanding of the attitude of stakeholders towards fish welfare and key factors that may affect fish welfare during live transport, using semi-structured interviews. This information will identify perceived and potential fish welfare issues for live transport in China and inform future research on this important issue that affects many animals each year. This preliminary study is part of a larger project that investigates the knowledge and attitudes of stakeholders and current practices for live transport of fish in the Chinese aquaculture industry. This report conforms to the Standard for Reporting Qualitative Research (SRQR) guidelines [35], with slight modifications.

## 2. Materials and Methods

A series of one-to-one interviews of Chinese stakeholders in the aquaculture industry (*n* = 12) was conducted between June and September 2020.

### 2.1. Participants

A purposive sampling strategy [36,41] was used to identify potential participants if they (i) were adults (at least 18 years of age) who spoke fluent Mandarin and (ii) had worked in the Chinese aquaculture industry or were involved with fishery-related jobs for at least one year (in total, even if they had changed their job or retired). We aimed to identify 1–2 participants from several job categories we identified. The initial recruitment had 13 people from within our existing network; however, one participant subsequently declined to be interviewed due to time constraints. Previous literature suggests an adequate sample size for qualitative research methods varies from 5 to 50 participants [42]. Text analysis was first carried out among the 12 participants to check saturation, which has been a “gold standard” for determining an adequate sample size in qualitative research [43,44,45]. Subsequently, no more recruitment was undertaken, and 12 participants in total were interviewed in this study.

Participants had different degrees of exposure to, or experience with, the aquaculture industry and held different fishery-related positions in China (Table 1). The twelve participants included nine males and three females. Nine participants lived in urban environments and three in suburban or rural regions. Various job categories across the aquaculture industry were represented, and some participants occupied more than one role. The three participants with the longest involvement in the industry (more than 15 years) all worked in fish sales. Three of the twelve worked at fish farms, two as employees (management positions) and one being self-employed (a farmer). Two participants worked in an animal protection/welfare organization as volunteers, one was a student who majored in ornamental fish rearing, and the other had experience with terrestrial animals and aquatic mammal protection. We also included two participants from fish research/academia. One of the remaining participants worked for a delivery and logistics company that delivered live and chilled aquatic products either by road or air. The last participant was a restaurant owner who sold live fish and seafood.

### 2.2. Interviews

Semi-structured interviews, lasting approximately 30 min each, were conducted individually either face-to-face (4) or by telephone (8), depending on the geographic distance and travel restrictions due to COVID-19. The lead researcher (Y.Y.) conducted the majority of interviews (11), and another volunteer conducted the remaining interview. Respondents participated in interviews based on an open-ended preliminary interview guideline (see Appendix A). Thirty-nine questions were included in that guideline, but participants were offered the opportunity to skip any questions or to indicate that they did not know or did not understand a particular question. All interviews followed a similar structure between individuals, with slight variations depending on their involvement in the industry. All interviews were conducted in Mandarin Chinese and audio recorded (iPhone7, Apple Inc., Cupertino, CA, USA). The recordings were subsequently transcribed into written Chinese by a second researcher (T.W.) and checked by the lead researcher, both of whom are fluent in Chinese. Notes were also taken during the interviews for evidence of emergent, preliminary concepts. Participants were asked to describe:

i. Their demographic information and employment status
ii. Their experience and knowledge on live transport of fish
iii. Variables that are monitored during live transport
iv. Factors that affect the success of live transport, as evidenced by fish mortality levels and fish well-being
v. Methods or criteria that are used to assess fish health after live transport
vi. Their understanding and opinion of “animal welfare” and whether this was a familiar term

### 2.3. Ethics

Ethical approval was obtained from the University of Queensland Human Research Ethics Committee before commencing the interviews (human ethics approval number: #2020/HE001290). Before each interview, participants reviewed and signed a participant consent form, indicating that the anonymity of the participant’s information would be retained and that participants had the right to withdraw from the study at their discretion.

### 2.4. Qualitative Analysis

Thematic analysis was aided by the use of NVivo 12 Plus software (QSR International Pty Ltd., Melbourne, VIC, Australia). The interview texts were coded automatically as nodes (questions) and cases (participants). The number of questions answered by each participant was counted, and questions that were answered by almost all of the participants (*n* > 10) were selected for analysis. Long answers with important emerging information or welfare- and transport-related terminology was extracted to identify themes and direct quotations (indicated by double quotation marks “…”) from participants’ conversations were used to interpret identified themes. Short answers such as “yes” or “no” were excluded from direct quotations as supporting evidence but were included in the analysis. Lastly, we grouped the 14 questions answered by all participants into three main themes: live transport processes, fish health and care, and animal/fish welfare. Capital letters (A–L) were randomly assigned to each participant as a unique anonymous identifier, and these appear with each quotation in the results section of the manuscript. Verbatim responses are displayed in quotation marks. In order to preserve the original meaning of the interviews, the transcript from each participant was first analyzed in Chinese and was subsequently translated into English by a bilingual volunteer. Subjects in Chinese language were often derived from the context of the direct quotes; they are added in parentheses for clarity. Chinese characters are also displayed with specific words mentioned by participants for a better understanding of both Chinese and Chinese-speaking researchers. Two researchers (Y.Y. and T.W.) reviewed the translations thoroughly for quality assurance and to ensure that the reports accurately conveyed the intention of the participants.

## 3. Results

### 3.1. Live Transport Processes

#### 3.1.1. Experience with Different Fish Species (Question 10)

The fish species that participants had experience with was dependent on their jobs. All participants indicated that they had worked with either freshwater fish, marine fish, or other aquatic animals. Freshwater fish species were the most commonly mentioned (*n* = 12). Among the freshwater fish, five participants reported that they had experience with the “four major Chinese carps” (四大家鱼): grass carp, silver carp, bighead carp (*Hypophthalmichthys nobilis*), and black carp (*Mylopharyngodon piceus*). Other freshwater fish species, such as crucian carp (*Carassius auratus*), mandarin fish (*Siniperca chuatsi*), and bass (*Lateolabrax japonicus*), were also mentioned, because many participants (*n* = 8) worked across several fish species groups. In addition to freshwater fish, four participants also worked with other live seafood, including marine fish and shellfish. Groupers were reported as one of the most popular seafood products in our study—for example, tiger grouper (*Epinephelus fuscoguttatus*). The two participants from aquaculture research/academia not only worked with commonly farmed fish species but also had experience with local fish species such as Amur minnow (*Phoxinus lagowskii*) or nationally protected wild fish, such as Tsinling lenok trout (*Brachymystax lenok tsinlingensis)* and Sichuan taimen (*Hucho bleekeri*).

#### 3.1.2. Transport Destinations and Their Areas (Question 11 and Question 12)

Participants were asked what sort of destinations they usually transported live fish to and where these places were located. They reported wholesale markets, supermarkets, wet markets, laboratories, restaurants, residential communities, and private residences as popular destinations. Wholesale markets were the most common destination mentioned (*n* = 6) for transporting food fish: (Participant A) “One of the most popular destinations is a large aquatic wholesale market” and (Participant E) “Fish are first transported to a wholesale market and they can subsequently be distributed to supermarkets or other shops.” In addition to markets, fish were also directly transported between fish producers and private buyers. According to participant A “[…] another [sales target] is the private buyer” and H “[…] there are some casual buyers, such as households from a residential community, we also deliver live fish to them.” Except for direct human consumption, fish are also transported alive for research purposes to laboratories. Participants D and G respectively noted, “We transport [wild-caught] fish from mountain areas to a laboratory” and “Fish are transported to our [university] laboratory for experiments.”

According to participants’ answers, the connecting and final destinations were mainly in urban areas (*n* = 8), with some in suburban areas (*n* = 2). Most destinations were reported to be provincial cities (e.g., Hangzhou, Changsha, and Changchun) or larger cities (e.g., Shanghai). Participant E said that large wholesale markets are gradually moving into suburban areas: “Nowadays, this sort of fresh market is to the extent possible near to the edge of the [Changsha] city, clustered between the city and suburb, where those transport trucks can get in and out.” The two animal advocates were not directly involved with live transport, but they stated that they purchase live fish in supermarkets and fresh markets, which are near to their (urban) residence (in Shenyang and Dalian). Live transport also occurs frequently within a province or between adjacent provinces, for instance, “[from a farm in Hangzhou to the transport destinations], within this area of Jiangsu, Zhejiang and Shanghai [up to 500 km approximately]” (Participant A) and “[live transport] usually occurs within the province, for example, from Jilin to Changchun [approximately 120 km], similar to that, maybe 2–3 h of trip” (Participant G).

#### 3.1.3. Proportion of Live Transport (Question 14)

Participants were asked how many fish are generally transported alive as a percentage of the fish transported in total. Their responses indicated that the live transport of fish in China is close to 100%, particularly for freshwater fish species. Most participants (*n* = 9) said that freshwater fish are mostly transported alive, for example, (Participant A) “From our experience, the fish transported are all alive, throughout the process of catching to putting into the transport tank” and (Participant G) “Fish all need to be alive for the transport.” While many marine fish species or other aquatic animals, such as crustaceans, can also be cold preserved (still alive) or directly frozen (dead) before transport, Participant J reported that “Our seafood here, it is live transported. There is also some seafood that is dead [due to capture], how is that transported? Under normal circumstances after being caught from the sea, they are directly frozen and iced [for transport].”

#### 3.1.4. Seasonal Effects on Live Transport (Question 15)

Interviewees were asked whether seasonal changes have an impact on the success of live transport. According to their responses, live transport occurs more in cool-weather conditions and less in summer, and the majority (*n* = 10) believed that seasonal changes have a great impact on the success of live transport. Water and ambient temperature were considered particularly important factors, for example, Participant A reported that “[…] when the ambient temperature is high, the water temperature will also be high, and the fish may experience hypoxia. So basically, the proportion of live fish transported in summer is very small.” Participants also suggested that fish mortality is higher in summer compared with other seasons. According to Participant G, “[…] under hot weather conditions, the [fish] survival rates are little lower. Then after they arrive at the destination, the fish condition apparently does not look good, and they need more time to recover, [during the recovery], even have a few deaths.” Similarly, Participant F stated, “[…] fish require constant water temperature during the whole transport [to survive]. In summer, you need to add ice [into the water]. In the winter you probably need to increase water temperature.”

In addition to the difference in ambient/water temperature, demand for fish species also varies by time of year and important events such as holidays or festivals: (Participant H) “This [live transport] will not change [due to seasons], it is mainly to do with workdays, or related to holidays, but for seasons, there is not that much relationship. […] for long-distance transport during holidays, the highway will be packed with traffic, and this is not good for live transport.” Live transport is also associated with fish production cycles throughout the year. Fish of different sizes and life stages are transported in different ways, as suggested by Participant C: “[…] in springtime, actually most transported fish are fry, then the rest in other seasons. Live transport of market size fish occurs more frequently.”

#### 3.1.5. Types of Transport Vehicle (Question 18)

During the interviews, we asked about what sort of vehicles are commonly used for live transport. Interviewees reported that the size and volume of the vehicle used in fish transport vary, dependent on transport purpose and distance. Commonly, purpose-built vehicles were used, as well as vehicles modified for live transport, such as trucks/lorries, vans, and private cars. According to Participant A, “Transport vehicles (活水车) must be professionally modified and equipped with oxygen supply, as well as water storage systems.” Modified trucks were typically used for transferring larger volumes of fish, as indicated by Participant F:” They are modified vehicles that have a tank with a circulation system, refrigeration and heating equipment.” In contrast, research fish can be transported using private cars because of their small volumes and smaller fish size. Participant G mentioned that “For fish transport vehicles, basically it is just us transporting them in the private cars that we drive, […] If we do not transport lots of fish, we just transport them back in our own private car[s]. Sometimes we find a van [for live transport] if there are more fish.”

#### 3.1.6. Transport Methods/Containers (Question 22)

Participants were asked about the most commonly used methods of transporting live fish. Based on their responses, transport tanks, plastic bags, and foam boxes were commonly used. Transport tanks made with polyvinyl chloride (PVC) plastic were commonly reported to be used for large volumes of fish or over long distances, with modified vehicles. For example, Participant A stated that “For large-scale transport, it has to be transport trucks with [PVC] tanks. For small-scale transports like we do, well, within a certain limit, we use plastic bags or foam boxes.” Participant E also mentioned: “Transport vehicles all use compartmented tanks made from that sort of PVC material, with oxygen added.” Small volumes of fish or short-distance deliveries were often carried out using aerated bags: (Participant G) “For transport within the province, about 2–3 h, fish are all in bags, fish transport bags.” Participant E also described that “If we are talking about after arriving at wholesale markets and transporting separately to grocery stores, or restaurants, then after arriving at wholesale markets they use foam boxes and aerated bags. At wholesale markets, they use that sort of foam box with aerated bags inside. This can also be used to transport live fish.” One participant (L) transported live fish regularly using a carrying pole (扁担). Fish were placed in homemade baskets with sealed plastic bags for transporting from his house to the local market on foot.

#### 3.1.7. Use of Fish Anesthetics (Question 24)

The use of chemical anesthetics in the live transport of food fish was not reported by participants in this study to be common. Most (*n* = 7) said that no fish anesthetics are used during live transport of food fish based on their experience, but other methods such as the addition of ice, a high concentration of sea salts, or vitamin C were applied to control water quality and reduce transport stress in the fish. Participant H: “We never use chemical anesthetics [for food fish], but sometimes we add a little vitamin C or sea salt.” The use of fish anesthetics seems to be species-specific in our study, evidenced by “There are some fish for which we will use anesthetics, such as yellow catfish because their body has a lot of spines and sharp bones” and “I know anesthetics have been used for marine fish. In terms of farmed fish, either freshwater or seawater, as long as they are relatively valuable fish types, they will usually use anesthetics”, from participants A and C, respectively. Two participants (D & G) answered that fish for research purposes were occasionally transported with anesthetics, as those are nationally protected species that are easily stressed by handling and transport processes.

#### 3.1.8. Loading and Unloading Processes (Question 25)

Question 25 obtained information on loading and unloading processes and their time points. According to most participants (*n* = 9), fish were caught by manual methods using dipnets or trawls. It is suggested by Participant A that “Fish are loaded and unloaded traditionally […] using a so-called ‘fishing net’ (拖网), or a fully sealed bucket with water, and [fishermen] will use that for loading and unloading.” Two participants mentioned that fish can be transferred by pumping and cage trapping. For example, participants C and J, respectively, stated that “One way is just directly to use a net to scoop up some fish […]. Another way, for relatively smaller fish, will be a sort of water pump that directly sucks the fish out” and “[…] in summer, you cannot pull fish out with a net. Generally, they use cages to trap fish, then to pull them up.”

Most stakeholders (*n* = 8) indicated that loading may occur several hours before transport starts, and fish were unloaded shortly after arriving at their destination. However, Participant F suggested that unloading may also occur at different times of the day, depending on traffic conditions and the opening hours of a market: “Fish do not have to be immediately unloaded [after arrival]. Basically, fish are unloaded in the afternoon or evening. Sometimes when the market has not yet opened, if a transport vehicle arrives [at the market] a little early, they will wait a bit. It is normal.”

#### 3.1.9. Knowledge around Live Transport (Question 34)

We asked all participants to self-rate their knowledge level and understanding in terms of live transport of fish on a five-point scale:

(i.) Very low level of knowledge/very poor understanding
(ii.) Low level of knowledge/not much understanding
(iii.) Moderate level of knowledge/general understanding
(iv.) High level of knowledge/rich understanding
(v.) Very high level of knowledge/very rich understanding

Five participants indicated they had a moderate level of knowledge/general understanding. Four participants said their knowledge level was low. Two reported a high level of knowledge/rich understanding, and only one participant self-rated a very high level of knowledge/very rich understanding.

### 3.2. Fish Health and Care

#### 3.2.1. Oxygen Supply during Transport (Question 26)

Participants were asked how oxygen was typically provided during live transport. Oxygen was considered necessary in both open and closed transport systems, according to participants’ answers. For an open transport system, liquid oxygen cylinders and air generators were used to provide dissolved oxygen (*n* = 11). Sufficient oxygen was aerated into plastic bags, e.g. by aerating from a liquid oxygen tank, when fish were transported in a closed transport system; therefore, no oxygen was supplied during live transport (*n* = 2).

#### 3.2.2. Challenges during Live Transport (Question 28)

Participants were asked to identify potential challenges that arise when transporting live fish. The control of water temperature, oxygen levels, transport density, water quality, vehicle vibration, the selection of transport distance, transport time, fish species, capture method, and driver experience were all mentioned by different participants as potential challenges during live transport. All participants identified more than one challenge during live transport, and water temperature was mentioned by 8 of the 12 respondents. The second most reported challenge (*n* = 7) was the maintenance of dissolved oxygen levels in transport containers. Transport density was also identified by three stakeholders, while transport time, fish species, travel time, and vehicle vibration were each mentioned twice or less in the study.

#### 3.2.3. Methods to Assess the Health Status of Fish (Question 31)

Fish outward appearance was a commonly reported indicator for assessing the health and marketability of transported fish. Key measures included the presence of bodily damage (injury or bleeding), a change of body color (red/white spots), eye color, and the secretion of skin mucus. For example, Participant A stated that “You must rely on experience to assess whether or not fish are healthy. You can observe whether or not fish bodies are descaling, whether they are bleeding, whether there are any red spots.” Similarly, Participant E reported: “Firstly, when fish arrive at their destination, one point is observing whether their eyes are clear (眼睛是否清澈), then the fish body, even checking whether their color is normal.” In addition to appearance, fish equilibrium, vigor, and swimming ability were also used to assess fish health after live transport. Participant D, for example, stated that they looked for whether fish showed a “floating head” (the fish ventral region was upward).

Assessment measures were reported to be more complicated for research fish, as evidenced by Participant G, “[…] we first observe the body color of the fish. Any fish that show abnormal body color will be excluded from our formal experiments. Furthermore, uneven fish sizes—too large or too small ones—we will not use them as well. For some high-value and expensive fish, we may do a microscopic examination to see whether they have any parasites, viruses or bacteria. […]. For the appearance check of fish, we only do simple checks, for instance, to see whether there is any body damage or mechanical injury to the fish.”

### 3.3. Animal/Fish Welfare

#### Understanding of Animal Welfare (Question 33)

Nine participants had previously heard of the phrase “animal welfare” and also were able to explain their understanding of the term. The remaining three did not know this term or were not sure of its meaning.

Some participants mentioned that pre-slaughter and slaughter conditions were influential for animal welfare, and animals should be treated well before or during slaughter. For example, “[…] animals should be treated as calmly as possible during slaughter” was suggested by Participant B. Some participants also believed that animal welfare affects product quality, in particular meat quality, as evidenced by Participant E: “[…] during the slaughter process, if animals experience that sort of excessive fear or shock, their bodies will produce acidic substances. It probably will result in worse meat quality, or fish quality. [animal] Welfare is probably just like this.” Some participants suggested that the husbandry and slaughter of farmed or aquatic animals should be standardized within China to improve animal welfare. They also indicated that animals should be kept in a good environment and that their mental state should be considered. Participant D suggested that “no matter the farming or processing practices, we should follow a principle that reduces pain and fear to animals.” Similarly, Participant G stated: “We should treat farmed animals as, how to say it, as humans. We should pay attention to their mental states and relevant issues. If we do not treat animals well during husbandry, they do not keep in a good mood or a good state. Their own condition can also affect their product quality.”

Three participants said that they were familiar with this term being applied to terrestrial livestock, but it was novel to describe fish or other aquatic animals in this way. For example, Participant A stated: “[…] it is quite fanciful for me that the term of animal welfare can be used in aquaculture.” Another participant (K) thought that aquatic animals did not have welfare, as she suggested that aquatic animals were not sentient like livestock and humans. This perspective differed between interviewees, as another participant (G) stated that fish should be treated the same as other farmed animals and that all food animals should have good welfare, regardless of the species.

The attitudes of stakeholders towards different animals could affect their welfare. Participant C gave an example that “[…] animal welfare is relative to our kinship distance (亲缘关系) and determined by the degree to which our lives are similar. As for non-food animals, for example, those ornamental fish, their welfare is probably a little better compared to food animals who are usually seen as food to prepare, so they probably do not have good welfare.” Animal welfare specifically in relation to transport was also mentioned by two participants. For example, Participant H mentioned that a low fish mortality rate meant better fish welfare during transport from a producer’s perspective. Participant E suggested that a good environment should be provided during transport.

## 4. Discussion

The results of this study are not fully representative of the entire Chinese aquaculture industry, but the findings provide insight into the industry in China and reveal fundamental information on fish welfare during live transport. We discuss live transport processes, fish health and care, and the animal/fish welfare implications of our findings.

### 4.1. Live Transport Processes in China

Our results confirm that live transport remains a key component of the Chinese aquaculture industry, in line with previous literature [14,46], particularly for freshwater fish. Various fish species were mentioned, including Chinese carps, which are dominant within Chinese freshwater aquaculture [13,15,47], and non-native fish species, such as tilapia, bass, and catfish [48,49]. Based on participants’ answers, both fish for food and research are transported alive in sealed containers or plastic bags using purpose-built trucks or private cars. Methods used for live transport are highly customized depending on transport volume, purpose, and fish species. Live transport occurs most frequently over short distances within a province (destined for a provincial city, up to 500 km approximately) or between adjacent provinces (up to 700 km approximately), based on the results presented in our study. Geographically, the Yangtze River region contains one of the highest concentrations of aquaculture producers in the central-eastern part of the country [11]. Participants from Zhejiang province also mentioned that live transport often occurs in this region between adjacent provinces: Jiangsu, Shanghai, and Anhui, all of which belong to the Yangtze River Delta, with an approximate distance from 170 to 460 km.

Participants indicated that fish are transported to various locations, but wholesale markets, often located in suburban areas, appear to be the most common commercial destination for food fish. Sales channels for aquaculture in China generally include producers, processors, traders (wholesalers), retailers, consumers, and restaurants [50]. Fish at wholesale markets can be directly sourced from individual farmers or from distributors who buy products from different farms [43]. Primary and secondary wholesalers are the main market operators who handle aquatic products before they reach smaller retailers, restaurants, and consumers [50]. Therefore, it seems that wholesale markets are an interim stop for live fish, and welfare concerns may still occur from a wholesale market to a final destination. In addition to large-volume transport, small volumes of fish are also transferred to private buyers for their home consumption [50].

Seasonal effects on the occurrence and success of live fish transport are substantial. Heat stress may be an influential factor on fish welfare during live transport, as most participants indicated that live transport occurs less frequently on hot weather days. Acute increases in water temperature are known to adversely affect fish physiology, with negative implications for fish welfare [51]. If fish must be transported in hot weather, then appropriate temperature reduction is necessary to maintain the welfare and survival of fish. For example, cooling of the transport water by 5–7 ℃, compared with the water from rearing systems, is a common protocol for live transport of salmon [44]. Methods such as ice cooling are often used to reduce water temperature during live transport [45], and these are similarly noted in the current study - the reported addition of ice into water. Lower water temperatures can also help to maintain water quality during transport [9]. For example, in a previous study, ammonia nitrogen concentrations were lower when largemouth bronze gudgeons (*Coreius guichenoti*) were transported in cool water [32].

Apart from water temperature, the maintenance of appropriate oxygen levels during live transport was also reported as one of the key controlling factors in our study. Sufficient oxygen supply is an essential component of the transport process [4,47]. Low dissolved oxygen levels not only alter fish behavior and physiology but also reduce their growth, and, more seriously, result in increased mortality [52]. In addition to these issues, participants also reported a variety of other potential challenges, many of which align with the previous literature, such as inappropriate transport density, transport time, poor handling and acclimation, and impact from mechanical vibrations [8,53,54].

From participant responses, it is clear that a variety of transport methods/containers are used to transport live fish, including transport tanks, plastic bags, and foam boxes. Plastic bags were noted to be more commonly used for the short-distance transport of a small volume of fish. This aligns with the literature from outside of China, which notes that market-sized fish are often transported in truck-mounted, high volume tanks over long distances [4,55], while smaller fish, such as juveniles and fingerlings, or some ornamental fish of high value, are transported in plastic bags [6,7,38,56].

From our interviews, chemical anesthetics were rarely used in live transport of most food fish but might occasionally be used for valuable fish (e.g., ornamental fish), research fish, and some food fish that are challenging to handle, although specific drugs were not mentioned in this study. Tricaine methane sulphonate (MS-222) is a common sedative that is approved as a food fish anesthetic by the US Food and Drug Administration (FDA), but it requires a 21-day withdrawal period before sale [57]. No anesthetics have been approved in China for fish handling and live transport of food fish [58]. The safety of aquatic products evokes concern from the public [58], and nowadays, there are more than 40 standards or regulations that control drug residual levels in aquatic products in China [59]. One previous survey reported that common fish anesthetics, including eugenol and isoeugenol, have been used for sedation of transported fish before being sold in Chinese markets, but the residual levels from investigated flesh samples were not high enough to be of risk to human health [58].

Capture, loading, and unloading methods noted in these interviews were still traditionally manual methods of moving fish, such as using dipnets. Chinese freshwater farming systems are typically carried out as a polyculture in pond systems, which enhances the utilization of all the available food resources in the pond ecosystem [12]. In this system, it is challenging to identify and only capture a single fish species without handling and manual sorting. Thus, the use of dipnets allows farmers to select specific fish species for their purposes. Another reason that traditional capture methods are still popular on Chinese fish farms could be the smaller scale of aquaculture companies. The proportion of small- and medium-sized aquaculture enterprises remains high in China, compared with European countries [59]. The costs of using advanced techniques on pre-transport procedures could be prohibitively expensive.

### 4.2. Fish Health and Care

Participants mentioned that the appearance, swimming ability, vitality, and mortality of fish were often checked after transport to estimate their health status and sale value. Physical damage to a fish looks unsightly and may lower the value of the fish [60]. Consumers often avoid unhealthy or injured fish because this indicates a lack of freshness [61]. In terms of the application to animal welfare science, these parameters are important outcome-based indicators that can be used to assess the welfare of individual fish, although the indicators are mostly species-specific [47]. Changes to body color are observable indices of welfare; for instance, they are observable stress indicators for salmon [24]. Changes in eye color and eye damage are also reliable and relatively easy welfare indicators from direct observation via the glass aquarium or checking individual fish on-site; however, they tend to be species-specific. For example, stressed Nile tilapia show eye darkening [62], while largemouth bass show corneal clouding after live transport [53]. Body injuries, including the presence of blood and loss of scales, are commonly used to check the health, welfare, and value of an individual fish [24,54]. Behavior can also be an observable indicator of fish health or welfare, although this may not affect fish marketability. Changes in fish behavior are considered welfare indicators. For instance, rainbow trout spend a longer time at the bottom of the tank, occupy a smaller number of tank sections, reduce swimming activity, and increase the number of abnormal movements after experiencing transport stress [63]. Variatus platy (*Xiphophorus variatus*), an ornamental fish, show increased occurrence of biting and freezing behaviors post-transport, which may increase the risk of injury [56].

### 4.3. Awareness and Understanding of Animal Welfare

This research provides insight into how stakeholders in Chinese aquaculture view animal welfare. Previous studies indicated that animal welfare is still in an early stage of development in China, reporting that about half of respondents had never heard of this term [64,65]. These studies mainly targeted the general public and showed that they are concerned for animal welfare because of its importance to food quality and safety [65,66,67]. Although industry professionals in our study also mentioned that animal welfare is related to product quality, they emphasized more heavily the connection between specific practices and animal welfare, for example, pre-treatment during slaughter and slaughter/handling standards. Therefore, the results from previous research with consumers may not be fully generalizable to industry stakeholders in the current study.

The concept of “animal welfare” has attracted growing attention within Chinese social media and researchers in recent years, although it was only introduced to mainland China in the 1990s [68]. There has been some positive transition in attitudes towards animals in China, which is due to economic development, increased concerns about food safety, and changes to human relationships with animals [43,69]. Food producers may therefore have more opportunities to be exposed to the concept of animal welfare, and their understanding could be enhanced by consumer feedback. This may give us some insight into why fish wholesalers and producers (farm managers) in our study were more familiar with the concept of animal welfare, as we expected. Consumers’ preferences for food safety is highly determined by socio-demographic variables in urban China [66]. A recent study suggests that Chinese urban consumers in large cities (e.g., Beijing or Shanghai) show a strong preference for choosing products that have good animal welfare and environmental stewardship, which is believed to be associated with better taste and safety of pork [70]. Therefore, education levels, concerns around food safety, and feedback between producers/retailers and consumers are potential ways to enhance positive attitudes towards the welfare of livestock and farmed fish.

Some participants felt that the concept of animal welfare refers more to the welfare of terrestrial animals, rather than fish. Currently, the main welfare focus for Chinese researchers, producers, and government authorities is to improve conditions for livestock, specifically for pigs [71,72] and chickens [73]. The publicity around the concept of animal welfare has also promoted the welfare of livestock in China, for example, the establishment of the International Collaborative Committee for Animal Welfare (ICCAW) in 2013, which is now the leading farm animal welfare organization in China [74]. This contributes to an increasing number of Chinese people, particularly younger generations, who show positive attitudes and behavior towards the welfare of livestock, as well as zoo animals [70,75,76,77]. These changes are also evident in the development of several relevant livestock standards. For example, the first enterprise-level welfare standard for dairy cattle was published by China Mengniu Dairy Corporation (Inner Mongolia Mengniu Dairy Group Co., Ltd., Hohhot, China) in 2020, which filled the gap in dairy cattle welfare management standards in China [78]. At legislative levels, national standards for the slaughter of livestock are now available and have been updated in China (GB/T 19479-2019).

However, farmed fish welfare has not received the same attention as terrestrial livestock from the general public and industry stakeholders in China. Legislation and welfare standards for farmed fish are already available in some European countries, while China as a major fish producer has paid little attention to fish welfare [28]. The recent developments for livestock welfare may be why some participants in our study felt that animal welfare referred to livestock species and not fish. The attitudes displayed by some participants towards fish welfare may in part be due to a lack of understanding of the needs of fish compared with other species. For example, one participant questioned whether fish are animals (like humans and livestock). Both in and outside of China, fish are often considered to be less important or less evolved than mammals [79,80]. For example, Callahan et al. [81] found that the American public ranked mammals as having the highest capacities of cognitive and emotional traits, while fish were ranked the lowest. Historically, it has been debated whether fish can suffer, although abundant evidence shows that fish are sentient and can consciously experience pain [79,82,83]. 

Fish are ectotherm (cool-blooded) animals that live in aquatic environments completely different to those of humans, and this difference may create barriers to understanding their feelings and welfare needs [47]. One participant mentioned that fish are less of a concern to them because of a lack of human–animal bond compared with land animals and the low genetic proximity between fish and humans. One study provided evidence to support this perception that mammals are the most emotive and cognitive species due to the “closeness” of their relationship with humans [81]. For some mammalian species, such as dogs and cats, people can observe and even interact with them. There may be a positive effect on these animals when interacting with humans because the social bond with the owner may be rewarding or the joint activities may be enjoyable in line with domain 4 in the Five Domains Model (behavior interactions) [21]. However, this bond is very challenging to achieve for most aquatic species, such as farmed fish who are part of a large production system [28]. It is also challenging to prepare welfare standards for each farmed fish species because of the diversity of species [28] and the polyculture systems on Chinese farms [12]. Currently, specialized welfare information is only available for around 20 species; therefore, the scientific literature on aquaculture welfare is still emerging and developing, and the issue needs on-going exploration [84]. Each fish species has different ecological and behavioral demands and varying physiological capacities, so information about one species cannot necessarily be translated to others. More effort is needed to develop appropriate standards for farmed fish in China, especially for polyculture systems.

### 4.4. Limitations

Participants in this study were a small cross-section of people specifically approached because they represented different stakeholders within the Chinese aquaculture industry. While we collected detailed answers from them in response to a range of free-text questions, the participants cannot be considered representative of the industry. The findings from this research will contribute to the development of a larger questionnaire that aims to be more representative in investigating fish welfare during live transport in China. Additionally, in this study, we not only aimed to investigate stakeholders’ attitudes towards animal welfare but also their knowledge on specific transport processes, to gain insight on this topic in China, as little is currently published. The two participants who were interviewed because of their role in animal welfare advocacy were not directly involved in fish transport and so were less likely to provide detailed contextual information compared with others who were directly involved with the process (e.g., retailers and farmers). However, they provided valuable insight into attitudes and perceptions around animal welfare. Therefore, a future study could be potentially developed to further examine similar insights from a range of non-industry stakeholders, such as the public and consumers, animal rights advocates, and aquaculture students. Although most participants indicated their willingness to participate in all the questions, one interview occurred in a market, and the participant was not able to answer all questions due to time limitations. The interview was interrupted by the participant’s business; therefore, the length of this interview was shorter than the rest.

## 5. Conclusions

Participants in this study confirmed that the live transport of fish (mainly farmed freshwater species) is common in the current Chinese aquaculture industry. Fish are transported using various methods and different vehicle types. The effect of seasonal changes on live transport is considered important, and transport is less commonly carried out in hot weather. Fish anesthetics are used to sedate some fish species but seldom used for food fish. Trawling and netting are still commonly used to catch fish from water due to the dominance of polyculture systems and many small-scale farms in Chinese aquaculture. Oxygen is always provided during live transport, and other challenges of keeping fish healthy are similar to those reported in the scientific literature. Physical appearance and fish vigor are used to measure fish well-being and marketability, according to participants’ answers. The self-estimated knowledge level of live transport was at a moderate level because most participants were not directly involved with live transport as a fish transporter. This study also provides information on the understanding of animal welfare and attitudes towards fish during live transport in China, as identified by a small sample of stakeholders in the industry. Although most stakeholders had heard of animal welfare and could provide their understanding, this term is considered more relevant to livestock or poultry, rather than fish. Future research should identify key welfare issues during live transport with a broader range of stakeholders and should investigate their attitudes towards fish welfare.

## Figures and Tables

**Table 1:** Demographic data of 12 participants (A–L) in China, from a survey investigating the knowledge and attitude of stakeholders in Chinese aquaculture industries on live transport of farmed fish.

Participant	Gender	Age Category	Education	Role in Industry	Years of Experience in the Industry	Residential Zone	Employment
A	Male	26–35	High school	Fish farm manager	4	Suburb	Employee
B	Female	36–45	Master’s degree	Animal welfare advocate	3	Urban	Volunteer
C	Male	18–25	High school	Animal welfare advocate	2	Urban	Volunteer
D	Male	36–45	Master’s degree	Fish researcher Government role relating to fishery	11	Urban	Employee
E	Male	36–45	No answer	Fish wholesaler (secondary)	16	Urban	Self-employed
F	Male	46–55	College	Fish wholesaler (primary)	15	Urban	Self-employed
G	Female	26–35	PhD degree	Fish researcher Aquaculture teacher	5	Urban	Employee
H	Male	26–35	College	Fish farm supervisor	4	Suburb	Employee
I	Male	26–35	Master’s degree	Fish transporter	5	Urban	Employee
J	Male	46–55	High school	Seafood restaurant owner	3	Urban	Self-employed
K	Female	36–45	No answer	Fish retailer	20	Urban	Self-employed
L	Male	Above 65	No formal education	Fish farmer	2	Rural	Self-employed

## Data Availability

The raw data is not available publicly or stored elsewhere due to ethical and privacy issues for all participants. Some anonymous data in this study can be requested from the corresponding author but will require the participant’s consent.

## Appendix A

### Semi-Structured Interview Guideline

1. What is your age range?
2. What is your gender?
3. What is your position in China’s aquaculture?
4. What is the highest education you have completed? What is your highest degree?
5. Which province in China do you work in?
6. What type of area are you currently living in? Rural, urban, suburban (where is your main residence)?
7. How do you acquire and maintain your work-related knowledge?
8. How long have you been engaged in the aquatic industry in China?
9. Can you describe your job?
10. What fish species do you work with?
11. Where are most live fish transported to?
12. Where are destination locations? More specifically: farm to market or town to town?
13. Normally how old are fish when they are transported?
14. What proportion of most fish are transported alive in China?
15. Do seasonal changes affect the success of live transport?
16. How many fish are transported alive each time?
17. What is the typical stocking density of live fish per truck?
18. What is the most common type of vehicle that is used to transport live fish?
19. Are the fish always kept in the same vehicle while being transported?
20. How long is the typical transport time?
21. Could you describe the road conditions during most of the live transport? Are they paved roads, unpaved roads, or a combination of two types?
22. Can you describe the typical methods of fish transport?
23. In general, what are the common procedures of fish monitoring during live transport?
24. Are there any anesthetics used in the live transport of farmed fish?
  - Yes/if yes, what is used?
  - No/not used.
25. What is the common loading and unloading process of live fish?
  A. In general, how long does the vehicle start to transport after the fish are loaded?
  B. In general, how long are fish unloaded upon arrival at the destination?
26. Is oxygen provided during live transport? If yes, how is it provided?
27. When the fish are delivered to your place, will you keep the fish alive for a few more days? How is their health condition?
28. What are the most challenging things to keeping fish in good conditions during live transport?
29. What is the average mortality rate of fish during or after live transport?
30. What do you think are the main causes of fish death during live transport?
31. How can you tell if a fish is healthy?
32. Do you know of any outbreaks of disease in the fishing industry in China? If yes, can you provide details of the disease?
33. Have you heard of the term “animal welfare”? If yes, how do you understand this concept?
34. How would you rate your knowledge/understanding of the live transport of farmed fish?
  - Very high level of knowledge/very rich understanding
  - High level of knowledge/rich understanding
  - Moderate level of knowledge/general understanding
  - Low level of knowledge/not much understanding
  - Very low level of knowledge/very poor understanding
35. Are you self-employed or employed by a company?
36. What do you like about your job?
37. Has the industry changed since you started working in it? Is there anything that can be improved in the industry?
38. Have you worked in aquaculture in other countries? If yes, which country? How is the industry in this country different from that in China?
39. In the aquatic industry, what factors do you think will affect buyers’ preferences?
